# Changes in operative trends and short‐term outcomes of surgery for congenital biliary dilatation in adults using real‐world data: A multilevel analysis based on a nationwide administrative database in Japan

**DOI:** 10.1002/ags3.12630

**Published:** 2022-10-18

**Authors:** Yasuhisa Mori, Makoto Okawara, Kazunori Shibao, Shiro Kohi, Toshihisa Tamura, Norihiro Sato, Yoshihisa Fujino, Kiyohide Fushimi, Shinya Matsuda, Keiji Hirata

**Affiliations:** ^1^ Department of Surgery 1, School of Medicine University of Occupational and Environmental Health Kitakyushu Japan; ^2^ Department of Environmental Epidemiology, Institute of Industrial Ecological Sciences University of Occupational and Environmental Health Kitakyushu Japan; ^3^ Department of Health Policy and Informatics Tokyo Medical and Dental University Graduate School of Medical and Dental Sciences Tokyo Japan; ^4^ Department of Preventive Medicine and Community Health, School of Medicine University of Occupational and Environmental Health Kitakyushu Japan

**Keywords:** congenital biliary dilatation, diagnosis procedure combination database, laparoscopic surgery, open surgery, real‐world data

## Abstract

**Aim:**

We aimed to evaluate the operative trends and compare the short‐term outcomes between open and laparoscopic surgery for congenital biliary dilatation (CBD) in adults using real‐world data from Japan.

**Methods:**

Data from the Japanese Diagnosis Procedure Combination database on 941 patients undergoing surgery for CBD at 357 hospitals from April 1, 2016, to March 31, 2021, were analyzed. The patients were divided into two groups: open surgery (n = 764) and laparoscopic surgery (n = 177). We performed a retrospective analysis via a multilevel analysis of the short‐term surgical outcomes and costs between open and laparoscopic surgery.

**Results:**

The rate of laparoscopic surgery has been increasing annually and had almost doubled to 25% by 2021. There were no significant differences in the in‐hospital mortality rate or postoperative morbidity between the two groups. The length of anesthesia was significantly longer in the laparoscopic than open surgery group (8.80 vs 6.16 hours, *p* < .001). The time to removal of the abdominal drain and length of hospital stay were significantly shorter in the laparoscopic than open surgery group (6.12 vs 8.35 days, *p* = .001 and 13.57 vs 15.79 days, *p* < .001, respectively). The coefficient for cost was 463 235 yen (95% confidence interval, 289 679‐636 792) higher in laparoscopic than open surgery (*p* < .001).

**Conclusion:**

The short‐term results were comparable between laparoscopic and open surgery for CBD. Further investigation is needed to validate our findings and long‐term outcomes.

## Graphical abstract

Our real‐world data highlight that laparoscopic surgery for CBD had short‐term results comparable to those of open surgery. The rate of laparoscopic surgery has been increasing every year; however, the number of cases is still low, and the high cost of laparoscopic surgery is an issue that requires further research to resolve. More high‐quality studies are needed to further validate our conclusions and long‐term outcomes.

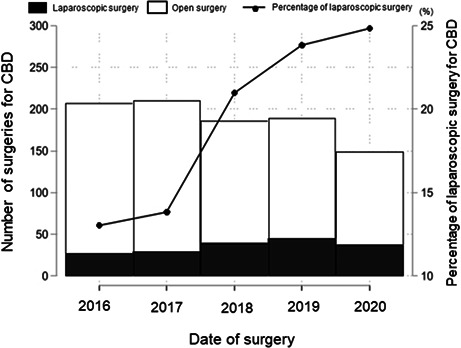

## 1 INTRODUCTION

Congenital biliary dilatation (CBD) has a higher incidence in Asia than in Europe and the United States and is three to four times more common in female than in male individuals.^1^ CBD is associated with a risk of malignant transformation of the dilated portion of the biliary tract and gallbladder because of its association with pancreaticobiliary maljunction, which causes pancreatic juice to reflux into the bile duct and cause chronic inflammation.^2^, ^3^, ^4^ Surgical management of CBD involves bile duct resection with cholecystectomy followed by biliary reconstruction.^5^, ^6^ Although the surgical procedure is relatively difficult among hepato‐biliary‐pancreatic surgeries because of the need to perform dissection of the intrapancreatic bile duct and hepaticoenteric anastomosis, the advantages of laparoscopic surgery for CBD include its safety, feasibility, and satisfactory short‐ and long‐term outcomes.^7^, ^8^, ^9^, ^10^, ^11^ Two meta‐analyses of laparoscopic versus open surgery for CBD in children have been reported^12^, ^13^; however, no meta‐analysis or randomized controlled trial of adults has been performed.

A health insurance system for all citizens that provides reimbursements in proportion to the total medical treatment fee has been in place since 1961 in Japan. Insurance reimbursements have covered laparoscopic surgery for CBD since April 2016 in Japan. The diagnosis procedure combination/per‐diem payment system (DPC/PDPS) of Japan for acute injuries or illness is a prospective payment system that uses a classification code based on the disease diagnosis and aims to standardize medical care, similar to the diagnosis‐related groups/prospective payment system, which was launched as part of the health insurance system in 2003.^14^, ^15^ The DPC includes inpatient data from an expansive list of hospitals throughout Japan and is assumed to reflect the real‐world circumstances within Japan’s medical fields.

In the present study, a multilevel analysis of real‐world data from the DPC database was performed to evaluate the operative trends and short‐term outcomes of surgery for CBD in adults after insurance reimbursements since April 2016 and compare the outcomes between laparoscopic and open surgery.

## 2 MATERIALS AND METHODS

### 2.1 Data source

This retrospective observational study was based on data from the DPC database, a Japanese administrative database and case‐mix system used as a tool to standardize medical profiling and payment. Of the approximately 1700 hospitals participating in the DPC database, 1540 institutions provided data during the study period. These data included disease names, costs, comorbidities on admission and during hospitalization coded according to the *International Statistical Classification of Diseases and Related Health Problems 10th revision* (*ICD‐10*), age, sex, body mass index (BMI), smoking status, length of postoperative hospital stay, medical procedures (including surgery), and discharge status (including in‐hospital death). We obtained these data from the DPC Study Group, which has data on 39 915 530 inpatient cases from 1540 hospitals from April 1, 2016, to March 31, 2021. The DPC Study Group, a government‐funded academic group, also collects copies of the DPC electronic data independently of the Ministry of Health, Labour, and Welfare for research purposes.^16^

### 2.2 Patients

The study population comprised inpatients who had been diagnosed with CBD (*ICD‐10* code Q444) from April 1, 2016 to March 31, 2021. The inclusion criteria were age ≥18 years, an inpatient status and admission to undergo open or laparoscopic surgery for CBD (K‐codes K674 and K674‐2, respectively). The exclusion criteria were patients with biliary cancer and missing data. The patients were divided into two groups according to their surgical procedure (laparoscopic or open surgery).

### 2.3 Endpoints

The following endpoints were selected to serve as surgical outcomes: in‐hospital mortality, length of postoperative hospital stay, reoperation with general anesthesia, use of antibiotics after 7 days postoperatively, transfusion within 7 days after surgery, unplanned postoperative reintubation, discontinuation of meals, postoperative morbidity, postoperative intervention (percutaneous or endoscopic drainage), and cost.

### 2.4 Statistical analysis

Associations between surgical outcomes and age groups were evaluated by multilevel regression models using a two‐level structure of individuals nested within the 357 institutions (multilevel analysis). Two‐level random‐intercept and fixed‐slope models were employed; these procedures consider independent violations among individuals in the same facilities and eliminate the possibility of the ordinary least‐squares estimator underestimating the true standard error.^17^, ^18^, ^19^ Investigating the contextual effects of laparoscopic and open surgery on surgical outcome requires adjustment for compositional individual factors. We used a multivariable model that included patient‐level factors that potentially correlated with outcomes. This model included the following possible confounding factors: sex, BMI, smoking status (current/ever or never), transfusion prior to surgery, duration of anesthesia, and number of surgeries for CBD during the observation period in the hospital to which the patient was admitted. We conducted multilevel logistic regression for binary outcomes and multilevel linear regression for outcomes that were treated as continuous variables, hospital codes being assigned to each hospital as a random effect. The STATA release 16 software program (StataCorp, College Station, TX, USA) was used for all calculations.

## 3 RESULTS

### 3.1 Patient characteristics

**FIGURE 1:**
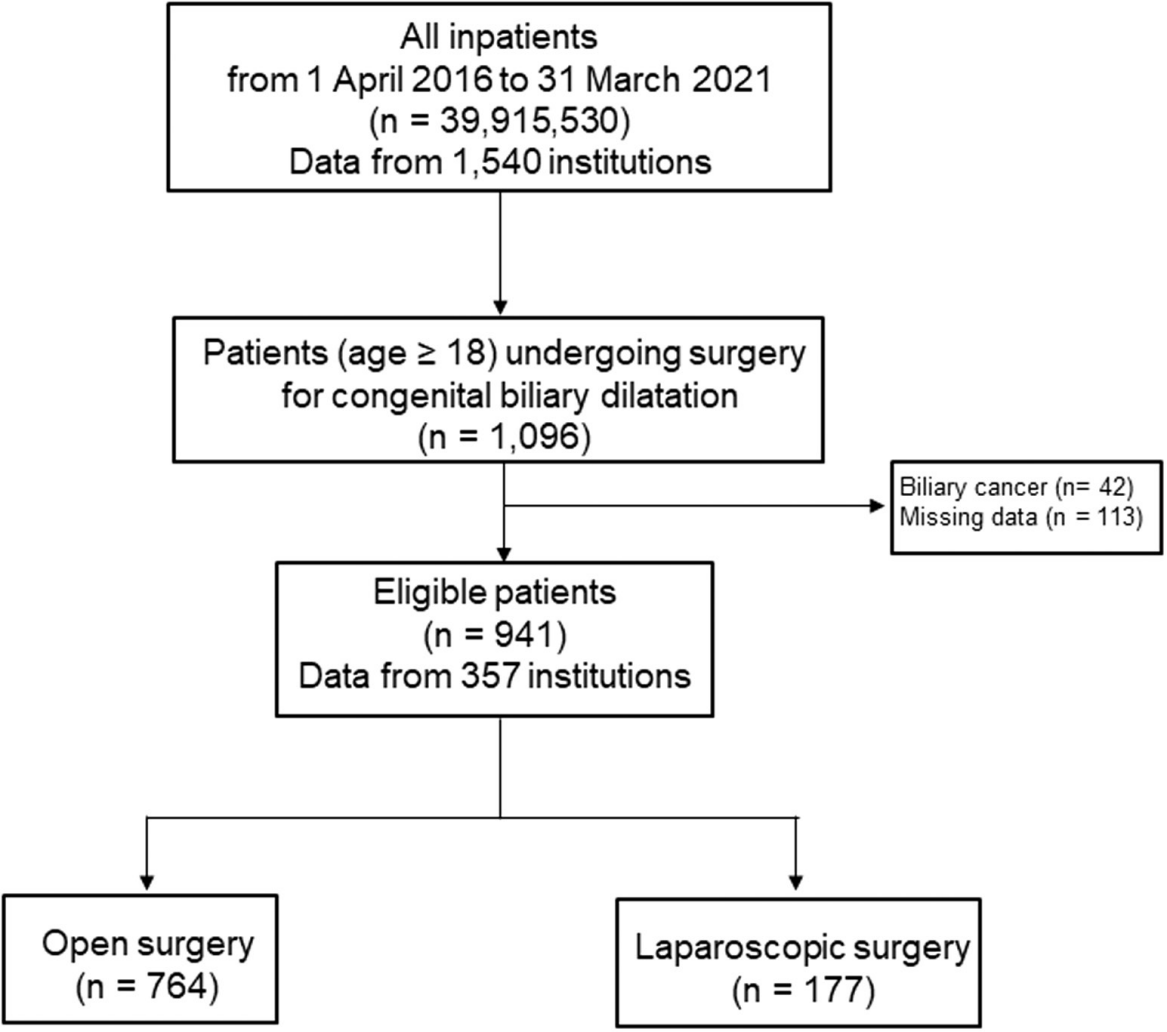
Flowchart of patient selection

Figure 1 is a CONSORT diagram of the participants drawn from the DPC database. The study cohort comprised 1096 patients who had undergone surgery for CBD from April 1, 2016, to March 31, 2021. Patients with biliary cancer (42 patients) and missing data for any variable (113 patients) were excluded. The remaining 941 patients who met the inclusion criteria were divided into the following two categories according to their surgical procedures: open surgery (764 patients) and laparoscopic surgery (177 patients).

### 3.2 Changes in operative trends

**FIGURE 2:**
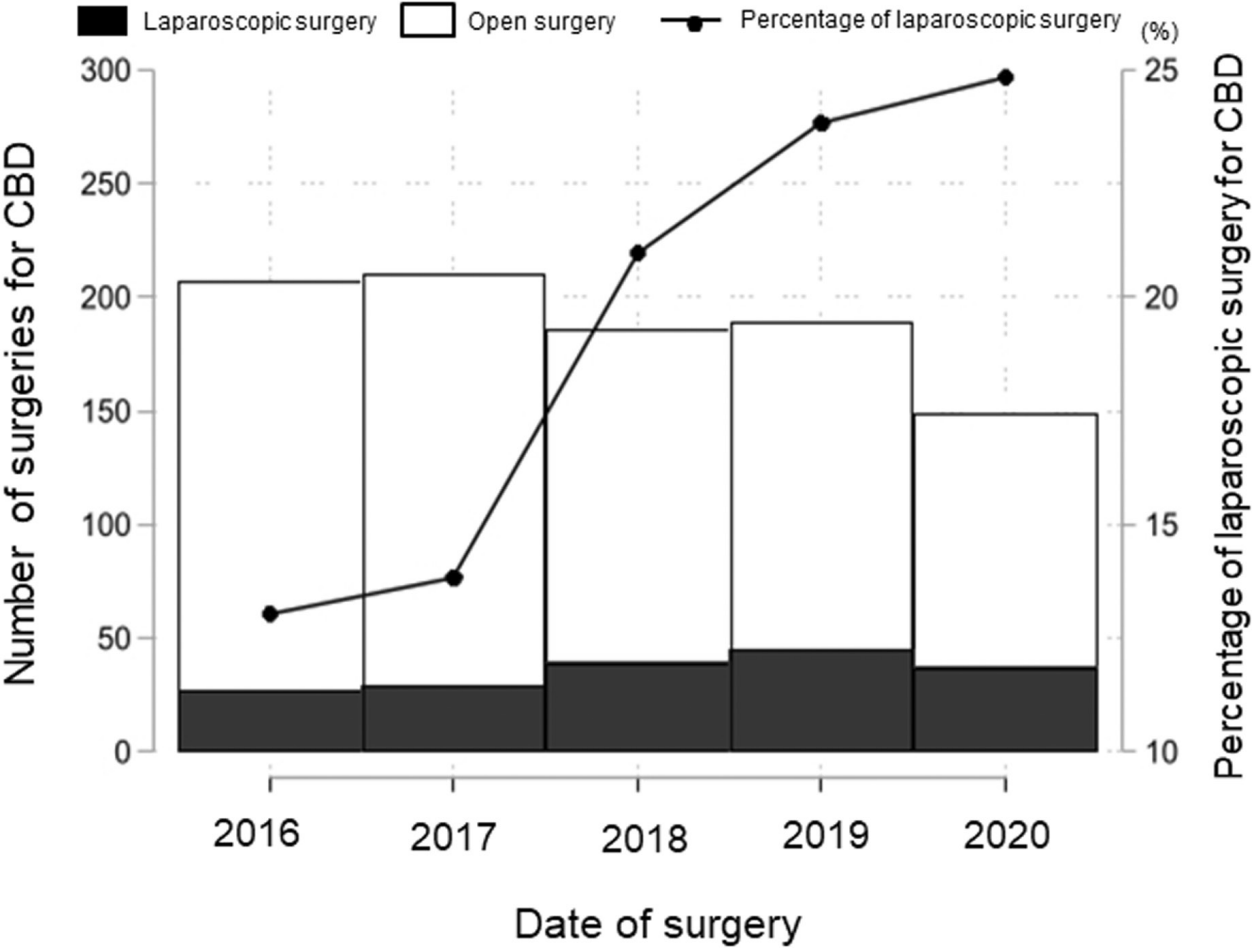
Changes in operative trends. CBD, congenital biliary dilatation

Figure 2 shows the changes in operative trends. Approximately 150‐200 surgeries per year were performed throughout the observation period. Since Japanese insurance began covering laparoscopic surgery for CBD in 2016, the rate of laparoscopic surgery, which was 13%, has been increasing every year and had almost doubled to 25% by 2021.

### 3.3 Basic characteristics of study participants

The basic characteristics of the study participants are shown in Table 1. CBD was approximately three times more common in women than men and was common in young people (48.2 ± 15.2 years of age). There were no tendencies regarding BMI, smoking status, or preoperative comorbidities between the two groups. Laparoscopic surgery tended to be performed more frequently than open surgery in hospitals in which relatively large numbers of surgeries for CBD were performed.

**TABLE 1:** Basic characteristics of the study population

	Total	Open	Lap
	n = 941	n = 764	n = 177
Variables			
Sex, female	711 (75.6)	562 (73.6)	149 (84.2)
Age, years	48.2 ± 15.2	49.1 ± 15.1	44.3 ± 15.3
BMI, kg/m2			
<18	62 (6.6)	54 (7.1)	8 (4.5)
18–25	644 (68.4)	510 (66.8)	134 (75.7)
>25	235 (25.0)	200 (26.2)	35 (19.8)
Smoking, current or former	240 (25.5)	200 (26.2)	40 (22.6)
Any comorbidity	145 (15.4)	123 (16.1)	22 (12.4)
Diabetes mellitus	104 (11.1)	88 (11.5)	16 (9.0)
Renal disorder	12 (1.3)	9 (1.2)	3 (1.7)
Ischemic heart disease	20 (2.1)	18 (2.4)	2 (1.1)
Heart failure	10 (1.1)	10 (1.3)	0 (0.0)
Cerebrovascular event	6 (0.6)	5 (0.7)	1 (0.6)
Respiratory disorder	2 (0.2)	2 (0.3)	0 (0.0)
Maintenance dialysis	2 (0.2)	1 (0.1)	1 (0.6)
Hospital‐level factor			
Number of surgeries for CBD within the study period			
1 or 2	318 (33.8)	288 (37.7)	30 (16.9)
3 or 4	174 (18.5)	146 (19.1)	28 (15.8)
5–8	218 (23.2)	176 (23.0)	42 (23.7)
9–21	231 (24.5)	154 (20.2)	77 (43.5)

*Note*: Data are presented as n (%) or mean ± standard deviation.

Abbreviations: BMI, body mass index; CBD, congenital biliary dilatation; Lap, laparoscopic surgery; Open, open surgery.

### 3.4 Postoperative outcomes

Table 2 shows the results of the association between the postoperative outcomes and surgical procedures. The in‐hospital mortality rate was similar between the open and laparoscopic groups (0.1% vs 0.0%, *p* = .630). Similarly, there were no statistically significant differences regarding reoperation with general anesthesia, use of antibiotics after 7 days postoperatively, unplanned reintubation, discontinuation of meals, postoperative morbidity, or postoperative interventions.

**TABLE 2:** Association between the postoperative outcomes and surgical procedures

	Open	Lap	*P*‐value
	n = 764	n = 177	
Outcomes	Events	Events	
In‐hospital mortality	1 (0.1)	0 (0.0)	.630
Reoperation with general anesthesia	15 (2.0)	6 (3.4)	.247
Use of antibiotics after 7 days postoperatively	188 (24.6)	42 (23.7)	.806
Transfusion within 7 days	36 (4.7)	6 (3.4)	.443
Unplanned reintubation	7 (0.9)	3 (1.7)	.363
Discontinuation of meals	163 (21.3)	30 (16.9)	.193
Postoperative morbidity	35 (4.6)	6 (3.4)	.484
Bile leakage	20 (2.6)	4 (2.3)	
Intra‐abdominal abscess	14 (1.8)	2 (1.1)	
Portal vein thrombosis	1 (0.1)	0 (0.0)	
Pulmonary embolism	1 (0.1)	0 (0.0)	
Renal failure	1 (0.1)	0 (0.0)	
Postoperative intervention	25 (3.3)	5 (2.8)	.760
Percutaneous drainage	20 (2.6)	3 (1.7)	
Endoscopic drainage	5 (0.7)	3 (1.7)	

*Note*: Data are presented as n (%) events.

Abbreviations: Lap, laparoscopic surgery; Open, open surgery.

Table 3 shows the results of the univariate and multivariate analyses of the associations between the postoperative outcomes and surgical procedures as estimated by multilevel logistic regression analysis. Compared with open surgery, the odds ratios for postoperative morbidity, transfusion within 7 days, reoperation with general anesthesia, and postoperative intervention of laparoscopic surgery were 0.73 (95% confidence interval [CI], 0.30‐1.77), 0.66 (0.24‐1.79), 1.89 (0.68‐5.29), and 0.86 (0.32‐2.28), respectively (all *p* > 0.05). Moreover, after adjustment for sex, age, BMI, smoking, coexisting diseases, and number of surgeries for CBD, the rates of all four postoperative outcomes were similar between the two groups.

**TABLE 3:** Mean difference in the postoperative outcomes estimated based on a logistic regression analysis

					Univariate				Multivariate^a^			
Outcomes	Surgical procedure	Surgeries (n)	Events (n)	Events (%)	OR	95% CI		*P*	Adjusted OR	95% CI		*P*
Postoperative morbidity	Open	764	35	4.6%	reference				reference			
	Lap	177	6	3.4%	0.73	0.30	1.77	.379	0.82	0.33	2.05	.670
Transfusion within 7 days	Open	764	36	4.7%	reference				reference			
	Lap	177	6	3.4%	0.66	0.24	1.79	.415	0.79	0.28	2.22	.652
Reoperation with general anesthesia	Open	764	15	2.0%	reference				reference			
	Lap	177	6	3.4%	1.89	0.68	5.29	.226	2.24	0.73	6.88	.159
Postoperative intervention	Open	764	25	3.3%	reference				reference			
	Lap	177	5	2.8%	0.86	0.32	2.28	.760	1.14	0.38	3.41	.810

Abbreviations: CI, confidence interval; Lap, laparoscopic surgery; Open, open surgery; OR, odds ratio.

a Estimated by a multilevel logistic regression model after adjustment for sex, age, body mass index, smoking, coexisting diseases, and number of surgeries for congenital biliary dilatation.

Table 4 shows the associations between the mean difference in the length of anesthesia, time to removal of the abdominal drain, time to starting meals, length of postoperative hospital stay, and cost as estimated by multilevel linear regression analysis. The length of anesthesia was significantly longer in the laparoscopic surgery group than in the open surgery group (8.80 vs 6.16 hours, coefficient 2.69, 95% CI, 2.34‐3.05, *p* < .001). However, the time to removal of the abdominal drain and length of hospital stay were significantly shorter in the laparoscopic surgery group than in the open surgery group (6.12 vs 8.35 days, coefficient −2.16, 95% CI, −3.44‐0.88, *p* = .001 and 13.57 vs 15.79 days, coefficient − 2.15, 95% CI, −4.11–0.19, *p* < .001, respectively). There was no statistically significant difference in the time to starting meals between the two groups. Compared with open surgery, the coefficient for cost was 463 235 yen (95% CI, 289679‐636 792) in laparoscopic surgery (*p* < .001). Notably, all of these calculations were performed after adjustment for sex, age, BMI, smoking, coexisting diseases, and number of surgeries for CBD, demonstrating that the length of anesthesia was longer (*p* < .001), time to removal of the abdominal drain was shorter (*p* = .003), and cost was higher (*p* < .001) in laparoscopic surgery than in open surgery.

**TABLE 4:** Mean difference in the length of anesthesia, postoperative recovery, and cost as estimated based on a linear regression analysis

				Univariate				Multivariate^a^			
Outcomes	Surgical procedure	Mean	Standard error	Coefficient	95% CI		*P*	Coefficient	95% CI		*P*
Length of anesthesia (hours)	Open	6.16	0.07	reference				reference			
	Lap	8.80	0.19	2.69	2.34	3.05	<.001	2.71	2.35	3.07	<.001
Time to removal of abdominal drain (days)	Open	8.35	0.27	reference				reference			
	Lap	6.12	0.36	−2.16	−3.44	−0.88	.001	−2.03	−3.36	−0.70	.003
Time to start of meals (days)	Open	3.34	0.08	reference				reference			
	Lap	3.35	0.16	−0.29	−0.66	0.09	.141	−0.17	−0.56	0.22	.399
Length of postoperative hospital stay (days)	Open	15.79	0.39	reference				reference			
	Lap	13.57	0.80	−2.15	−4.11	−0.19	.031	−1.12	−3.11	0.87	.269
Cost (yen)	Open	1 637 203	27 134	reference				reference			
	Lap	2 101 226	132 060	463 235	289 679	636 792	<.001	479 251	303 659	654 843	<.001

Abbreviations: CI, confidence interval; Lap, laparoscopic surgery; Open, open surgery.

a Estimated by a multilevel linear regression model after adjustment for sex, age, body mass index, smoking, coexisting diseases, and number of surgeries for congenital biliary dilatation.

## 4 DISCUSSION

The three main findings of the present study are as follows. First, although the length of anesthesia was longer in laparoscopic surgery than in open surgery, the short‐term results of laparoscopic surgery were comparable to those of open surgery. Second, since Japanese insurance began covering laparoscopic surgery for CBD in 2016, the rate of laparoscopic surgery has been increasing every year. Third, the hospital cost of laparoscopic surgery was higher than that of open surgery.

The rate of diagnosis of CBD in adults, which is estimated at only 20%, is reportedly lower than that in children.^20^ Several studies, including meta‐analyses, have therefore focused on the postoperative outcomes between laparoscopic and open surgery for CBD in children^12^, ^13^; however, few such studies of adults have been reported. Because CBD is a rare disease, we collected data on a large number of patients using the DPC database to compare postoperative outcomes, including costs, between laparoscopic and open surgery in the current study. To the best of our knowledge, this study involved one of the largest sample sizes of adult patients with CBD. Although the DPC database does not include detailed intraoperative information including the operative time, the length of anesthesia, which was substituted for the operative time in this study, was longer in laparoscopic surgery than in open surgery. Meanwhile, although there was no significant difference in postoperative morbidity between the two groups, the time to removal of the abdominal drain and the length of hospital stay were significantly shorter in the laparoscopic surgery group than in the open surgery group. One possible reason is that laparoscopic surgery can be attributed to a faster recovery of bowel peristalsis compared with open surgery, which may lead to the earlier removal of an abdominal drain and an earlier discharge from hospital. Alternatively, it may reflect the lower rates of antibiotic use after 7 days postoperatively, transfusion within 7 days, the discontinuation of meals, and postoperative intervention in the laparoscopic surgery group, despite no significant differences in these outcomes occurring between the two groups. Previous reports support our results regarding these operative outcomes.^21^, ^22^, ^23^, ^24^ Because CBD is a benign disease that is common in young female patients, an optimal cosmetic outcome is desirable, and laparoscopic surgery can be considered safe and feasible for CBD in these patients.

The present study revealed that the rate of laparoscopic surgery was relatively low at 25% in 2021, although it had been increasing every year after insurance coverage began. One possible explanation for this is that the surgical procedure is relatively difficult among hepato‐biliary‐pancreatic surgeries because of the need to perform dissection of the intrapancreatic bile duct, which is especially challenging in cases of severe inflammation due to preoperative cholangitis, and to perform hepaticoenteric anastomosis. Another possible explanation is that high proximal dissection should be performed to resect the stenotic segment whenever possible, especially in patients with Todani type IV‐A CBD^25^, and two‐duct anastomosis may be necessary; these procedures are often technically difficult for surgeons during laparoscopic surgery, even in experienced centers. To solve these issues, robotic surgical systems can overcome the above‐mentioned limitations by their articulated instrumentation, tremor filtering, motion scaling, and three‐dimensional high‐definition view. With the recent advent of robotic surgical systems, the safety and feasibility of robot‐assisted surgery for CBD are being increasingly reported.^26^, ^27^, ^28^ Insurance reimbursements have covered robot‐assisted surgery for CBD since 2022 in Japan. Although robotic procedures are not yet standard in Japan, further technological improvements and device development are expected.

Ziogas et al^29^ reported that laparoscopic liver resection is associated with higher operative costs than those of open liver resection for major hepatectomies; however, this is offset by a lower hospital cost for laparoscopic than open liver resection, resulting in similar total cost. With respect to surgery for CBD in Japan, the operative cost is 1 100 000 yen for laparoscopic surgery but 594 900 yen for open surgery. In the current study, although the time to removal of the abdominal drain and length of postoperative hospital stay were shorter in the laparoscopic surgery group, the coefficient for total cost was approximately 470 000 yen higher after adjustment for patient‐ and institution‐related factors. As is expected with new technology, cost will remain high until the technology is widely accepted and adopted into routine clinical practice. Although the breakdown of the hospital cost could not be extracted from the DPC database in the present study, this discrepancy may reflect the dissimilarity in reimbursement systems among different countries. It may also be a consequence of surgeons spending more time and effort in performing the operation, with a subsequent increase in cost for laparoscopic surgery.

Although the findings of our epidemiological research using the DPC database reflect the current status of treatment of CBD in Japan, this study had some limitations. First, despite the large number of patients with a rare disease, this was an observational and retrospective study. Second, the DPC database lacks information on several relevant patient‐related factors, including laboratory findings, the Charlson comorbidity index, and the Todani classification,^25^ which are important considerations when planning treatment for CBD. In addition, the database lacks information on details of the surgical procedure, operative quality, and indicators of surgical difficulty such as the duration of the procedure. Third, patients undergoing surgery for CBD are at risk of developing late complications such as stricture of hepaticojejunostomy, cholangitis, hepatolithiasis, liver abscesses, and cholangiocarcinogenesis of the remnant bile duct. Thus, careful long‐term postoperative follow‐up is necessary after the operation; however, the DPC database only includes data of hospitalized patients, preventing analysis of long‐term outcomes. Further investigation using other real‐world data is necessary to clarify the effects of CBD on long‐term outcomes.

In conclusion, our real‐world data highlight that laparoscopic surgery for CBD had short‐term results comparable to those of open surgery. The rate of laparoscopic surgery has been increasing every year; however, the high cost of laparoscopic surgery is an issue that requires further research to resolve. More high‐quality studies are needed to further validate our findings and long‐term outcomes.

## DISCLOSURE

Funding: This study was funded by the Ministry of Health, Labour, and Welfare, Japan (grant number: 20AA2005).

Conflict of Interest: The authors declare no conflicts of interest for this article.

Author Contribution: All authors substantially contributed to the conception and design of the study. Dr. Okawara and Dr. Fujino contributed to the data acquisition and interpretation. Dr. Mori drafted the manuscript, and all authors critically revised it for important intellectual content. All authors gave final approval for this version to be published.

## ETHICAL STATEMENTS

This study was conducted with the approval of the Ethics Committee of the University of Occupational and Environmental Health (Approval No. R2‐007). The procedures conformed to the provisions of the Declaration of Helsinki. Because of the retrospective study design, the requirement for written informed consent was waived. The protocol has been published on our home page (http://www.uoeh‐u.ac.jp/kouza/1geka/intro_j.html) in accordance with the guidelines for research ethics of the Ministry of Education, Culture, Sports, Science, and Technology and the Ministry of Health, Labour, and Welfare of Japan (http://www.lifescience.mext.go.jp/bioethics/ekigaku.html).
